# Effectiveness of Group Dialectical Behavior Therapy (Based on Core Distress Tolerance and Emotion Regulation Components) on Expulsive Anger and Impulsive Behaviors

**DOI:** 10.5539/gjhs.v6n7p116

**Published:** 2014-09-18

**Authors:** H. R. Jamilian, A. A. Malekirad, M. Farhadi, M. Habibi, N. Zamani

**Affiliations:** 1 Department of Psychiatry, Arak University of Medical Sciences, Arak, Iran; 2 Department of Biology, Payam Nour University, Tehran, Iran; 3 Department of Psychology, Hamedan Buali Sina University, Hamedan, Iran; 4 Family Research Center, Shahid Beheshti University, Tehran, Iran; 5 Azad University, Higher Education Campus, Hamedan, Iran

**Keywords:** dialectical behavior therapy, distress tolerance, emotion regulation components, expulsive anger, impulsive behaviors

## Abstract

**Introduction:**

The purpose of this study is to measure Effectiveness of group dialectical behavior therapy (basedon core distress tolerance and emotion regulation components) on Expulsive Anger and Impulsive Behaviors.

**Materials & Methods:**

Research method is a semi experimental socio-statistic approach consisting of experimental group (dialectical behavior therapy) and control group. Participants were patients referred to Amir Kabir Hospital in Arak who suffered from Expulsive Anger and Impulsive Behaviors. Based on stratified random sampling, 16 patients (women) were placed in each group. Research tools included the structured diagnosis interview according to DSM-IV-TR (2000), Barrat impulsivity scale (1994) Distress Tolerance Scale (2005) Difficulties of Emotion Regulation Scale (2004) and dialectical behavior therapy were done for two months,8 group-sessions).

**Findings:**

Dialectical behavior therapy was effective on Expulsive Anger and Impulsive Behaviors.

**Discussion & Conclusion:**

Distress tolerance and emotion regulation components were effective on Expulsive Anger and Impulsive Behaviors.

## 1. Introduction

Many people commit dangerous behaviors in their daily life (Moeller et al., 2001). Impulsive behavior and explosive anger are generally known as dangerous behaviors (Zamani, Farhadi & Jamilian, 2012), and encompasses a wide range of immature and pleasure seeking behaviors, which are usually accompanied by high risk (Ekhtiari, 2005) Impulsiveness is the main cause of many social problems such as uncontrolled sexual behaviors, drug abuse, personality disorders, and crime (Evenden, 1999).

Impulsiveness and aggressive impulsive behaviors without programming and thinking beforehandare too risky (Swann & Hollander, 2002) and impulsive people suffer disorders of judgment (Miller, Rathus, & Linehan, 2007). Explosive behaviors among impulsive people are in spite of explosive behavior among impulsive control disorders in which people feel helpless and tendency to take an action, superficial inability to resist tendency to do irrational and dangerous behaviors. In the former case, people show explosive behaviors to free themselves from stress (Halgin & Whitborne, 2002)

Explosive disorder emerges as separate periods of lack of impulsive and aggressive lack of control, which in most of the cases lead to serious attacks to oneself or others. The extent of aggressiveness is effective on initiation of such periods; a symptom that the patient may express it in words and take few minutes to several hours. Regardless of the length of this period, the state is not permanent and after the peak, the individual regret what they had gone through (Millon, 2002). Eysenck (1993) argued that impulsiveness and explosive behaviors are unintentional risk taking behaviors that are part of neurotics (Weiss, 1996) and main features of borderline personality disorders (BPDs).

To show the extent of the issue of anger and hazardous behavior it is enough to mention that 1000 people commit suicide each day in the world. This figure has grown 60% in the last 45 years, which is a warning as committing suicide is the worst outcome of impulsive behavior and explosive anger that follows a growing trend in many countries. This problem is known as the worst issue in mental health field and 10^th^ of September is named by WHO as the Day of Fighting Suicide. (Koons et al., 2001) Any record of committing suicide increases the chance of doing it up to 40 times (Hawton, 2000) and suicide among impulsive and explosive personalities as a request for help (Dickman & Meyer, 1988) These patients are not able to delay their emotions and suffer problems in controlling their behavior and decision-making. These problems leads to suicide by taking aluminum phosphide, cyanide, or self-immolation, or hanging oneself and other sudden not-programmed actions to express oneself and ask for others’ support.

Increase in number of suicide has led to introduction of several medications. However, and in spite of the fact that many research works support effectiveness of the proposed medications, there is not a 100% treatment for the problem to be called as the definite solution for dangerous and self-damaging behaviors. The problem is that such behaviors are negative emotional impulses that happen intentionally or unintentionally. In addition, dangerous and self-damaging behaviors are one of the 9 criteria of diagnosing BPD, although, impulsive people without the rest of BPD symptoms cannot be categorized in this category. In addition, a new diagnostic classification has been defined to encompass impulsive personalities by “mental disorders statistics and diagnostic guideline” revised 4^th^ edition.

Extensive works are carrying out to propose new theories and treatments for people suffering BPS. American Association of Psychology published the guidelines for treatment of BPS for the first time. The key points of this guideline are detection of multi-aspect nature of pathology of borderline, proposing a flexible treatment approach that meet demands and expectation of the patients, emphasis on combined approach and combining medication with psychotherapy and conditions for admitting and looking after hospitalized patients (Oldham, 2006). Considering surveys carried out thus far, there are three treatment guidelines regarding BPD, which have been criticized, including psychotherapy treatments, mental pharmacology, and clinical treatment. On the other hand, accidental clinical behavior therapy and some treatments such as dialectical behavior therapy have been supported.

Dialectical behavior therapy is one of the novel treatment approaches, which is based on cognitive behavioral approach and was first introduced by Marsharlinhan (Linehan et al., 1991) for BPS treatment. Dialectical treatment method is a technique based on accepting the patients as they are (mindfulness and sustaining distress as acceptance elements), while they are helped to make change in their behavior by receiving trainings regarding interpersonal skills and emotions regulating skills (Davison, Neal, Kring, & Johnson, 2007)

Effectiveness of dialectical behavior therapy for treatment of these disorders have been proved by Ketz et al. (2004), Konzo et al. (2001), Bohus et al. (2000 & 2004), Pasieczny and Connor (2001) in the field of BPDs and Mcquillan et al. (2005). Miller et al. (2007) regarding people with suicide tendencies and Mckay (2007) regarding patients who committed violent crimes.

In addition, surveys of effectiveness of dialectical behavior therapy in attenuating impulsive behaviors among women suffering from BPD showed no significant improvement, although trivial improvements were observed. Many works have shown that dialectical behavior therapy is effective in treating impulsive behavior by Soler et al. (2009) and Mcquillan (2005).

Taking into account epidemy of dangerous behaviors (impulsive behaviors and explosive angers) among patients who show impulsive behaviors but are not diagnosed with BPD and that the study population is suitable for surveying new behavioral cognitive approaches (concerning cognitive level and being open to novel treatment approaches) justify necessity and soundness of the present study. Among the elements of dialectical behavioral therapy, two of them (resisting distress as an element of acceptance and regulating emotions) as the elements in attenuating the symptoms were studied. The present study, therefore, is an attempt to answer whether the treatment methods of BPD are effective on impulsive behaviors and explosive angers (as the features of BPD) or not?

## 2. Methodology

The present study is semi-experimental applied work with control and experiment groups designed as pretest and posttest. Study population was the referrals to Amir Kabir Educational Treatment Center, Arak city and constituted of women. The inclusion criteria were minimum education level of associates’ degree; age range of 25-35; no medicine medication throughout the study; gaining SD + 1 point in Barratt Impulsive Scale, DTS, and DERS. In Karbalaei et al. (2011) research, group therapy was carried out on 8 members group. Following (Karbalaei et.al, 2011) intervention in this study was carried out through group therapy and the participants in the experiment group (n = 16) were divided into two groups of 8; control group was also comprised of 16 members. Average age of the participants in the experiment and test groups was 30.62 ± 2.4 and 29.81 ± 3.06.

### 2.1 Research Tool

Research tools included clinical interview, Barratt Impulsive Scale, distresstolerance scale, hardship of distress regulation scale, and dialectical therapy behavior trainings.

Clinical interview based on diagnostic and statistical guidelinesof mental disorders: structured and semi-structure diagnostic interview including a systematic set of specialized questions, which are aimed to assess a set of behavioral patterns, thoughts and emotions of the referrals. It is relevant to disorder diagnosis to some extents (Karbalaei et al., 2011).

Barratt Impulsive Scale: the questionnaire is a proper tool to measure different types of impulsive behaviors. The 11^th^ edition of the questionnaire was developed by Prof. Ernest Barratt (Barratt, Stanford, Kent, and Felthous, 2004). It comprises of 30 questions (four-alternative questions) that cover three factors of cognitive impulsiveness (cognitive decision-making) motor impulsiveness (thoughtless actions), lack of programming (measured based on lack of forethought). The structure of the questions represents aspects of accelerated decision-making and lack of forethought (maximum point = 120). The questionnaire has positive significant correlation with Aisne Impulsivity questionnaire- indication of criterion validity.

Farsi translation of Barratt impulsivity scale was done by Ekhtiari et al and reliability and validity were satisfactory. Reliability was obtained 0.83, while the reliability of the English version of the questionnaire was reported by Barratt equal with 0.81, and that of Italian version was 0.79. Cronbach’s alpha was obtained between 0.4 and 0.83 (Ekhtiari, Rezvanfard, & Mokri, 2008)

Distress tolerance scale (DTS): a self-report index of emotional distress tolerance with 15 items and 4 subscales. The subscales include tolerance (emotional distress tolerance), attraction (attraction to negative emotions), assessment (mental assessment of distress) and regulation (regulation of measures to sooth distress). Cronbach’s alpha of the scales were 0.72, 0.82, 0.78, and 0.70 respectively and total α of the scale was 0.82. In addition, criterion reliability and preliminary correlation were acceptable (Simons & Gaher, 2005). The questionnaire was used for Iranian population for the first time by Alavi et al. in Iran. Total internal consistency reliability of the scale (a = 0.71) was high and reliability of the subscales was moderate (0.58) (tolerate (0.42), attraction (0.56), assessment (0.58), and regulation (0.58)) (Alavi et al., 2011)

Difficulties of emotion regulation scale (DERS): a self-report scale to measure emotional mal-regulation in a more comprehensive form relative to the available tools in this field. The scale is comprised of 36 elements and six subscale including non-acceptance, difficulties of engaging goal-directed behavior (goals), impulse control difficulties (impulse), lack of emotional awareness (awareness), limited access to emotion regulation strategies (strategies) and lack of emotional clarity (clarity). Higher points indicate more difficulties concerning emotions regulation (Gratz & Roemer, 2004)

The results of reliability check by Gratz and Romer showed high internal reliability of the scale (total reliability of the scale (a = 0.93) and subscales non-acceptance (a = 0.85); goals (a = 0.89); impulse (a = 0.85), awareness (a = 0.80); strategies (a = 0.88); clarity (a = 0.84)). In addition, reliability of test and retest for total point of the scale was acceptable (a = 0.88; p<0.01). The scale was used for Iranian population by Alavi et al. (2011) for the first time and high internal consistency for the total scale was confirmed (a = 0.86) (Gratz & Roemer, 2004)

Dialectical behavior therapy: one of the novel treatment approaches based on behavior cognitive approach, which was first introduced by Marshalinhan for treating BPD. Indeed, dialectical behavior therapy is an approach that combines referral-basedacceptance and solidarity with cognitive behavior and social skills trainings. It is comprised of four interventions including group structured treatment sessions (skill learning), private session, telephone consultation with consultant, and experts team sessions to support dialectical behavior therapist (Zamani & Habibi, 2014).

### 2.2 Research Method

Following interview with psychologist, the participants filled out Barratt Impulsivity Scale, DTS, and DERS as pretest. Afterward, dialectical behavior therapist examined the participants. The control group received no intervention. Dialectical behavior therapy was designed based on Matthew Mc K, Jeffery Wood, and Jeffery Brenthly’s following Linhan’s book “Skill Learning” (Mckay, Wood, & Brantley, 2007), as an 8 sessions program (each skill 4 session). The treatment was started with acceptance-based skills (distress tolerance skills) –the centerpiece of the treatment method–given that the patients suffered from dangerous behaviors and distracting and troublesome emotions. Afterward, treatment was followed by change-based skills (emotional regulation skills).

Two first sessions of distress tolerance skills was dedicated to basic skills of tolerating distress and distraction (fundamental acceptance, distracting from self-damaging behaviors, joyful activities, concentration on work or other issues, distracting, distracting by leaving the situation, distracting by assigning daily works, distracting by counting, setting distraction programs, self-calming, and codifying resting design). In the next two sessions, advanced skills were under focus including (visualizing a safe environment, discovering values, detecting super powers, living at the moment, using self-motivating defensive thoughts, basic acceptance, self- confirming utterance, and setting defensive approaches). The 5^th^ and 6^th^ sessions were focused on the basic skills of emotional discipline including (detecting emotions, how emotions act, removing obstacles of healthy emotions, reducing damages caused by distressing emotions, self-monitoring, cutting cognitive vulnerability, and improving positive emotions). Finally, the last two sessions were about advanced skills of emotional regulation including intentional concentration on emotions instead of judging them, dealing with emotions, acting despite intense emotions, and problem solving.

## 3. Findings

The study was carried out on36 female participants divided into control and experimental groups (n = 16). Age range of the participants was 25-35 and average age of the experimental and control groups was 30.62 ± 2.4 and 29.81 ± 3.06. T-test showed no significant difference between the two groups concerning age average (t = 0.77, p = 0.44). Minimum education degree was associates’ degree (25% of the control group, 12.5% of the of the control group). In addition, 43.8% of each group were holder of Bachelors’ degree (highest frequency) and 18.7% of the experiment group and 31.2% of the control group were holder of Masters’ degree. Regarding occupation of the participants, the results indicated that 31.2% of the control group and 37.5% of the control group were housewives, while 62.5% of the control group and 68.8% of the experiment group had job outside the house. Therefore, employed participants were the majority in the both groups. Concerning marital status, 18.8% of the both groups were unmarried and the rest were married (81.2%) (Table 1). Economic condition and number of children were not under consideration.

**Table 1:** Demographic comparison of the control and experiment groups

		Experiment group	Control group
		n(%)	n(%)
education	high-school diploma	(12/5)2	(12/5)2
	associates’ degree	(25)4	(12/5)2
	bachelors’ degree	(43/8)7	(43/8)7
	Masters’ degree	(18/7)3	(31/2)5
occupation	housewife	(31/2)5	(37/5)6
	employed	(68/8)11	(62/5)10
marital status	unmarried	(18/8)3	(18/8)3
	married	(81/2)13	(81/2)13
age	ave.	62/30	2/84
	SD	81/29	06/3

Given that the present study is of repetitive measurements design, data analyses were carried out using variance analysis test with repetitive measurement and t-test for consistent samples. Variance analysis with repetitive measurements including the participants’ point before intervention and after intervention as internal factor of the subjects (dependent variables) and the groups (experiment and control) as the moderate variables was input to the model (Table 2). The results of the test were used for surveying the changes of the three variables of impulsive behavior, explosive angers, distress tolerance, and emotion regulation before and after interventions.

**Table 2:** Variance analysis and repetitive measurement

Dependent variables		df (factor)	df (error)	f	p
impulsive behaviors and explosive anger	group	1	30	3/67	0/021
	time	2	60	11/02	0/011
	group * time	2	60	5/09	0/002
distress tolerance	group	1	30	6/02	0/017
	time	2	60	9/02	0/09
	group * time	2	60	6/47	0/017
emotion regulation	group	1	30	2/04	0/03
	time	2	60	5/89	0/000
	group * time	2	60	13/07	0/07

As indicated in Table (2), the results of variance analysis and repetitive measurements concerning effectiveness of dialectical behavior therapy onimpulsive behaviors and explosive anger showed significant interaction between time and group.

To determine accurate nature of this difference, post hoc t-test was used on the consistent samples (Table 3) and as indicated by the results, impulsivity and explosive anger points of the participants in experiment group were significantly decreased after intervention (SD = 7.02; M = 94.56) comparing with before intervention (SD = 6.07; M = 65.01). On the other hand, impulsiveness points of the participants in the control group were not significantly different before and after the intervention.

**Table 3:** Standard deviation and average of the points and t-test for test and control group before and after intervention

Questionnaire	group	Average (SD) before intervention	Average (SD) after intervention	t value	p
impulsive behaviors and explosive angers	experiment	94/56(7/02)	65/01(6/97)	6/78	0/000
	control	83/56(14/99)	78(13/28)	5/46	0/069
distress tolerance	experiment	41/30(5/31)	27(2/25)	7/89	0/089
	control	43/05(5/91)	41/09(6/13)	5/78	0/062
emotion regulation	experiment	103(15/07)	89(11/98)	-2/08	0/013
	control	101(14/97)	93/13(14/53)	1/04	0/032

## 4. Discussion and Conclusion

Pretest and posttest points clearly indicated reduction of impulsive behaviors points after dialectical behavior therapy (pretest: 94.56 and posttest: 65.01). This reflects effectiveness of dialectical behavior therapy. Indeed, the intervention is actually designed for people that show self-damaging behavior such as wounding oneself, thinking about suicide, tendency to suicide, and committing suicide (Lynch et al., 2003). Linhan prescribed this method for the patients with acute disorders (e.g. suicide, life threat to others, life threatening behaviors such as reckless driving, uncontrolled sex behaviors, and overeating), impulsive patients, and those suffering from uncontrolled behaviors. The clinical psychiatry section of the American Association of Psychology confirms the intervention as an effective treatment for BPD (Alilo & Sharifi, 2011)

Impulsivity is one of the key features and symptoms of the disorder, which starts in early age of adulthood and prepares the ground for explosive anger and further disorders. Zanarini (Alilo & Sharifi, 2011) recommended classifying BPDs as impulsive control behaviors. Furthermore, according to Millon’s classification known as “impulsive borderline personality” (Alilo & Sharifi, 2011), which is a combination of histrionic or anti-society patterns, these patients are excessively impulsive and irresponsible and are mainly diagnosed with uncontrolled negative emotions and cognitively disordered. In addition, the patients demonstrate failure to regulate positive emotions, which leads to irrational and shallow excitement. Since these patients are not capable of thinking power, they cannot have programmed behavior or take into account consequences of their actions when they try to free themselves from social requirements and obligations. One may conclude then, that the key to success of dialectical behavior treatment was attenuation of the problem of engaging with emotional problems and the skills taught to the patients reduces unwanted emotions including impulsivity.

Linehan (1991) in “effectiveness of dialectical behavior therapy” showed that the patients that received dialectical behavior therapy were more successful in regulating and controlling their emotions and reported less anger and anxiety. In addition, these patients were more successful concerning interpersonal functions and social compatibility. Other studies by Ketz et al. (2004), Koons et al. (2001), Bohus et al. (2000, 2004) Pasieczny and Connor (2001) on BPD, and Mcquillan et al. (2005). Miller et al. (2007) on the patients who committed violent crimes confirmed effectiveness of dialectical behavior therapy.

In addition, Clarkin et al. (2007) showed in their works that dialectical behavior therapy is effective in soothing anger, and Soler et al. (2009) confirmed effectiveness of the treatment to deal with impulsive behaviors. Moreover, Miller et al. (2007), Vanden Bosch et al. (2002) showed that dialectical behavior therapy was effective in attenuating impulsivity, self-damage regulating emotions, and improving behavioral/emotional problems such as depression, anxiety, anger, emotional instability, and emotionality.

Given the results, skill training helps the patients to reach emotional stability and improve interpersonal characteristics; as they feel the need of being accepted by others. Indeed, these patients are over-aggressive because of being rejected by others and unfulfilled needs in their interaction with nurses and others who look after them. These people always move between dependency and independency so that they are led to unstable relations, feeling emptiness, seeking over-intimate relations, and ambivalence. Interpersonal skill training helps determining communicational style, increase of relevance between the patient and others’ needs, and determining personal relations and problems in this regard (Soler et al., 2009).

Lack of normalized tool to measure explosive angers, limited time, necessity of permanent monitoring, and gradual transfer of skills were some of the limitations of the present study. Furthermore, the results can be generalized for the patients of explosive anger and impulse behaviors who seek treatment and undergo long-term treatments. It is notable that only women participated in the study and therefore, the results can be generalized to women suffering from impulsive behaviors who seek medication. To have more accurate results of effectiveness of the intervention, future studies can bring male patients to the study or employ the intervention to treat improper emotions. Given the positive results of the intervention among female patients, the treatment can be a part of a long-term treatment program for impulsive patients.
